# Treating Opioid Use Disorder With Methadone in Pharmacies

**DOI:** 10.1001/jamanetworkopen.2026.0703

**Published:** 2026-03-16

**Authors:** Cynthia A. Tschampl, Sage R. Feltus, Elena Soranno, Jeffrey Bratberg, Maureen T. Stewart, Murray Dawson, Traci C. Green

**Affiliations:** 1 The Heller School for Social Policy and Management, Brandeis University, Waltham, Massachusetts; 2 Department of Health Law, Policy & Management, Boston University School of Public Health, Boston, Massachusetts; 3 Department of Pharmacy Practice, University of Rhode Island College of Pharmacy, Kingston

## Abstract

**Importance:**

Methadone, a medication for opioid use disorder, is underused in the US response to the opioid overdose crisis. Under federal law, methadone can be offered in pharmacies via medication units, although a change to federal statutes or regulations could allow more options for pharmacists to dispense methadone.

**Objective:**

To analyze innovative return on investment (ROI) for 2 pharmacy-based models: (1) pharmacy-based medication unit and (2) pharmacist-dispensed methadone.

**Design, Setting, and Participants:**

This cross-sectional economic evaluation, conducted from March 13, 2024, to August 22, 2025, created 2 models for pharmacy-based methadone dispensed at community-based pharmacies in the US to people who are stable on methadone maintenance treatment and who were referred by the opioid treatment program (OTP) or who recently initiated treatment due to the new option for pharmacy dispensing: (1) an OTP and a pharmacy partner to operate a medication unit in the pharmacy wherein OTP clinicians prescribe and pharmacists dispense methadone and (2) regulatory changes that allow medical professionals to prescribe methadone and pharmacists to dispense the medication. Each model included startup costs plus 3 years of operational costs and revenue. Input data were extracted from key informant interviews, a time-motion study, and the literature. Data were analyzed from June 2024 to July 2025.

**Exposures:**

Models addressed clinical services, anticipated consumers, potential competitors, key differentiators, and critical success factors. Input data were combined with microcosting methodology and enumerated minimum, best, and maximum values (PertBeta distributions) for 121 key inputs. A total of 10 000 Monte Carlo simulations were conducted to obtain 95% uncertainty intervals (UIs). Values are presented in 2024 US dollars.

**Main Outcomes and Measures:**

The primary study outcomes were ROI ratios and net profits after 3 years in operation.

**Results:**

The medication unit model (model 1) assumed a total of 3429 (95% UI, 1385-6244) client visits over the 3 years, indicating a mean (SD) of 95 (36) visits per month. For the pharmacist-dispensed model (model 2), the total number of client visits was estimated as 793 (95% UI, 531-1167), indicating a mean (SD) of 22 (5) visits per month. For model 1, over 3 years, a pharmacy would net $3.53 (95% UI, $1.14-$6.99) for every $1.00 spent, yielding a profit of $96 904 (95% UI, $5365-$267 451), including a 93.8% likelihood of netting $15 000 by year 3. For model 2, over 3 years, a pharmacy would net $2.64 (95% UI, $2.04-$3.41) for every $1.00, yielding a profit of $23 844 (95% UI, $15 045-$36 546), including a 97.6% likelihood of netting $15 000 or more by year 3.

**Conclusions and Relevance:**

In this economic evaluation of 2 pharmacy-based methadone business models, both models demonstrated positive ROI over 3 years. This finding suggests that increased access to methadone—a key step in reducing overdose deaths—can be profitable for community pharmacies. Changes to federal statutes or regulations may be needed, depending on the preferred model.

## Key Points

**Question:**

What is the estimated return on investment for a pharmacy-based medication unit or for medical professional–prescribed and pharmacist-dispensed methadone?

**Findings:**

In this economic evaluation, the pharmacy-based medication unit model returned $3.53 per $1.00 spent and a net profit of $96 904 over 3 years. The pharmacist-dispensed methadone model returned $2.64 per $1.00 spent and a net profit of $23 844 over 3 years.

**Meaning:**

The findings of this study suggest that expanding access to methadone for opioid use disorder through a pharmacy-based model is financially viable for pharmacies.

## Teaser

This economic evaluation conducts return on investment analyses for 2 pharmacy-based models of treating opioid use disorder with methadone: (1) a pharmacy-based medication unit and (2) pharmacist-dispensed methadone.

## Introduction

Opioid overdose is an ongoing public health crisis in the US. Methadone is 1 medication for opioid use disorder,^1^ and treatment with methadone is strongly associated with decreased risk for fatal overdose^2^ and all-cause mortality.^3^ Under current federal law and regulations, methadone may be administered or directly dispensed only by federally certified opioid treatment programs (OTPs) on an ambulatory basis, with rare exceptions (eg, hospitals may provide 72-hour treatment while arranging referral to an OTP).^1,4,5^ There are approximately 2100 OTPs in the US, but 80% of counties and the entire state of Wyoming lack even 1 OTP.^6,7^ Barriers to accessing methadone treatment at OTPs include transportation difficulties,^8,9,10,11^ limited availability in rural communities,^12^ long waiting lists,^13^ not allowing children on site or offering childcare,^14,15^ and limited clinic hours.^16^ People with opioid use disorder (OUD) receiving methadone treatment, patient advocacy groups, addiction treatment clinicians, health care professional organizations, and federal policymakers have all advocated for expanding access to methadone through retail or community pharmacies.^16,17,18,19,20,21,22^

Community pharmacies in the US are well suited to offer medications for opioid use disorder and other clinically relevant services. Pharmacists have been dispensing methadone for pain indications for decades. Distance from home to treatment at an OTP is associated with treatment retention,^23,24,25^ and some rural residents have to drive close to 2 hours per day for methadone treatment.^26^ In contrast, almost 50% of rural census tracts have a community pharmacy within a 20-minute drive.^27^ Pharmacies currently fill buprenorphine and naltrexone prescriptions for OUD and dispense naloxone for prevention of fatal overdose.^28,29,30^ Independent pharmacies, which are more common than chain pharmacies in rural locations that also lack OTPs,^31^ are interested in the opportunity to provide pharmacist-dispensed methadone.^22^

Without changes to the Controlled Substances Act, there are at least 2 ways that methadone for OUD could be offered in US pharmacies. First, pharmacies could partner with OTPs to operate satellite OTP locations that dispense methadone for OUD in the pharmacy, known as a *medication unit model*.^1,4^ Medication units are not common, and there are barriers to establishing them, including lack of knowledge of the option, unclear and prohibitive federal regulations, state laws banning medication units, and financial viability.^6,32^ Second, pharmacists in the US could administer or directly dispense methadone for OUD if the Drug Enforcement Administration (DEA) explicitly permitted qualified practitioners to prescribe methadone to be filled at pharmacies, an act that represents a broadening of the interpretation of statute 21 U.S.C. 823(h) but no change to federal law.^1,4,33,34^ A new law to amend the Controlled Substances Act could also allow this approach. This second option is referred to as the *pharmacist-dispensed model*.

The implementation of pharmacy-based methadone requires financial sustainability. We conducted a return on investment (ROI) analysis from the perspective of a pharmacy engaging in the medication unit model (model 1) and the pharmacist-dispensed model (model 2). ROI analyses answer the question of whether a business activity will make a profit or a loss, and to what degree.

## Methods

This cross-sectional economic evaluation was conducted from March 13, 2024, to August 22, 2025. The Brandeis University institutional review board approved this study. We followed the Consolidated Health Economic Evaluation Reporting Standards (CHEERS) reporting guideline.^35^

### Overview of Delivery Models

In strategy 1, the medication unit model, OTPs and pharmacies form cost-sharing partnerships that allow the OTP to operate a satellite methadone dispensing site at the pharmacy. The pharmacist operates as a part-time staff member for the OTP, receiving methadone for OUD from the OTP and dispensing it at the pharmacy. The client receiving methadone remains a client of the OTP and returns to the OTP for ancillary services while picking up methadone at the pharmacy-based medication unit with frequency determined by the OTP.

In strategy 2, the pharmacist-dispensed model, a pharmacist dispenses methadone for OUD prescribed by a DEA-registered practitioner and orders, tracks, stores, and disposes of methadone through the same systems that they have in place for other controlled substance medications. The person with OUD is under the care of a DEA-registered medical clinician who is responsible for any ancillary services and ensures compliance with federal regulations. eTable 1 in Supplement 1 displays key model details.

### Data Sources

To identify key components required for the ROI models, we obtained insights from people who use methadone in various ways. First, we reviewed the Liberating Methadone Conference Report^17^ and conference recordings, from which we identified key considerations that applied to both models (eg, need for access beyond OTPs, pharmacist training in OUD treatment, and a private space for observed dosing). Second, following the COBRE (Centers for Biomedical Research Excellence) Principles for Community Empowering Research,^36^ we conducted 2 in-person community advisory board meetings in June 2024 with individuals in long-term recovery from OUD with experience accessing methadone through OTPs (n = 3) and individuals in early recovery and/or current drug users who had experience with methadone or buprenorphine treatment (n = 5). After obtaining verbal informed consent, we briefly presented an outline of the medication unit strategy and the possibilities of a pharmacist-dispensed strategy to participants. We asked participants open-ended questions about preferred dosing schedules, pharmacy hours, privacy considerations, services they would want to see offered at the pharmacy, stigma, and infrastructure considerations. We deidentified and transcribed the audio recording to facilitate identification of additional components relevant to both models (eg, a desire for pharmacy staff to undergo antistigma training and not requiring urine toxicology testing at the pharmacy). Additional details are in eMethods 1 in Supplement 1. To further identify startup and operational costs, we conducted interviews with OTP leadership (n = 5), pharmacy leadership (n = 7), private and public payers (n = 6), and state and federal policymakers (n = 5); these participants provided verbal consent. We used a combination of purposive and snowball sampling to recruit individuals. Interviews took place from April to July 2024 via videoconference for 40 to 60 minutes.

We searched the literature and commercial websites to extract values for cost and revenue inputs not specifically obtained through interviews. We used the US Bureau of Labor Statistics to ascertain staff wages.^37^ To help calculate labor costs, we conducted a time-motion study,^38^ simulating tasks relevant to both models while recording and timing the tasks (eTable 2 and eMethods 2 in Supplement 1). We obtained final outstanding inputs by consulting an independent pharmacy owner (M. Olivier, PharmD, via notes after a telephone call, June 16, 2024).

### Statistical Analysis

Data were analyzed from June 2024 to July 2025. We applied microcosting methodology^39^ to the data that we collected to estimate the cost and revenue values shown in Table 1^37,40,41,42,43,44,45,46,47,48,49,50,51,52,53,54,55,56,57^ and Table 2.^58,59,60^ Additional methodological details are provided in eMethods 1 and 2 and eTables 3 to 6 in Supplement 1.

**Table 1.** Startup and Annual Costs for the Pharmacy-Based Medication Unit and Pharmacy-Dispensed Methadone Models

Model	Labor inputs across periods	Minimum^a^^,^^b^	Best	Maximum	Source
1	Lawyer hourly wage, $	53.91	87.69	121.13	US Bureau of Labor Statistics,^37^ 2023
1, 2	Pharmacist hourly wage, $	48.29	64.85	77.63	US Bureau of Labor Statistics,^37^ 2023
1, 2	Pharmacy technician hourly wage, $	14.27	21.34	27.65	US Bureau of Labor Statistics,^37^ 2023
1-SA	Security guard hourly wage, $	20.62	10.19	29.05	US Bureau of Labor Statistics,^37^ 2023
1, 2	No. of pharmacists	1	1	4	Authors’ estimate per key informants
1, 2	No. of pharmacy technicians	0	1	11	Authors’ estimates per key informants
**Model startup costs**					
Additional labor inputs					
1	Pharmacist hours: set up parallel storage and management systems	16	24	40	Authors’ estimate; US Bureau of Labor Statistics,^37^ 2023
2	Pharmacist hours: set up new standard operating procedures	1	3	7	Authors’ estimate; US Bureau of Labor Statistics,^37^ 2023
1	Lawyer hours: review of contract with the OTP	5	10	24	Authors’ estimate; US Bureau of Labor Statistics,^37^ 2023
Material inputs					
1	Single-drawer filing cabinet with a lock for disposal, $	96	161	265	Litfad; Global Industrial; Uline^40,41,42^
1	High-security DEA-recommended safe, $	130	361	645	Grafco; Graham-Field; Uline; Global Industrial^43,44,45,46^
1-SA	Liquid methadone dispensing machine, $	650	897	7841	LabDirect; Sigma-Aldrich; Parker Hannifin^47,48,49^
1-SA	OTP label printing machine, $	92	344	525	Barcode Factory; Brother; Supplyline^50,51,52^
1-SA	Telemedicine tablet device, $	230	319	349	Apple^53^
1-SA	Telemedicine headset, $	13	20	30	Logitech^54^
1, 2	Overhead costs applied to all startup costs, %	5	12	15	Authors’ estimate
**Model annual costs^c^**					
Ongoing clientele inputs					
1	No. clients per month, year 1/year 2/year 3^c^	5/7/8	9/13/15	15/21/25	Author estimate; NSDUH^55^
2	No. clients per month, year 1/year 2/year 3^c^	6/8/10	10/15/17	17/24/29	Authors’ calculation (15% increase over model 1)
1	No. visits per client, all years	4	1	28	Authors’ estimate per key informants
2	No. visits per client, all years	1	1	4	Authors’ estimate per key informants
Ongoing labor inputs					
1	Pharmacist hours per year (eg, OTP coordination, troubleshooting new systems)^d^	6	12	20	Authors’ estimate
1	Pharmacist hours per client visit, year 1/year 2/year 3	0.09/0.09/0.06	0.12/0.10/0.08	0.21/0.18/0.16	Authors’ time-motion study
1-SA	Pharmacist hours per client visit, year 1/year 2/year 3	0.12/0.11/0.08	0.15/0.14/0.14	0.42/0.40/0.37	Authors’ time-motion study
1	Pharmacy technician hours per client visit, year 1/year 2/year 3	0.03/0.03/0.02	0.05/0.05/0.04	0.06/0.06/0.05	Authors’ time-motion study
1-SA	Pharmacy technician hours per client visit, year 1/year 2/year 3	0.03/0.03/0.02	0.05/0.05/0.04	0.06/0.06/0.05	Authors’ time-motion study
2	Pharmacist management hours per year (eg, PAs and dosing coordination)^d^	5	11	15	Authors’ estimate
1, 2	Pharmacist hours: additional training and compliance	1	2	7	Authors’ estimate
2	Pharmacist hours per client visit, year 1/year 2/year 3	0.11/0.10/0.10	0.19/0.17/0.16	0.26/0.24/0.23	Authors’ time-motion study
2	Pharmacy technician labor hours per client visit, year 1/year 2/year 3	0.03/0.03/0.02	0.04/0.04/0.03	0.05/0.05/0.04	Authors’ time-motion study
Ongoing material inputs^e^					
1	DEA medication unit license (annualized), $	296	NA	NA	US Congress^56^
1, 2	Methadone wholesale acquisition cost, $^f^	0.00	NA	NA	Accounted for in profit line
1-SA	Alcohol wipes for sanitation, $	0.02	NA	NA	CareTouch^57^
1, 2	Overhead applied to all recurring costs^g^	5	12	15	Authors’ estimate

Abbreviations: DEA, Drug Enforcement Administration; NA, not applicable; NSDUH, National Survey on Drug Use and Health; OTP, opioid treatment program; PAs, prior authorizations; SA, scenario analysis.

^a^ Pert-Beta distribution was used for all inputs with a minimum and maximum listed.

^b^ All monetary amounts are shown in 2024 US dollars.

^c^ Year 1 values shown. We further estimated there would be 40% growth in year 2 of operation and an additional 20% growth in year 3 of operation, where clientele would stabilize. See additional details in eTable 3 in Supplement 1.

^d^ Year 1 values shown; assuming efficiencies would develop over time, we used 90% of these values for year 2 and 80% of these values for year 3.

^e^ We considered translation services as a cost but did not add it assuming pharmacies already have a service they access and would incur no additional costs.

^f^ The methadone wholesale acquisition cost is subtracted from the profit line. A key informant provided the amount in this manner.

^g^ See additional details and inputs in eMethods 1 and 2 and eTables 2 to 6 in Supplement 1.

**Table 2.** Inputs to Calculate Revenue for the Pharmacy-Based Medication Unit and Pharmacy-Dispensed Methadone Models

Item description	No. delivered per year^a^			Revenue per unit, $^b^			Source
	Minimum	Best	Maximum	Minimum	Best	Maximum	
Model 1							
Monthly medication unit payment from OTP to pharmacy^c^	NA	12	NA	1379	1724	2068	Authors’ estimate; Centers for Medicare & Medicaid Services^58^
Monthly medication unit rent	NA	12	NA	342	414	1026	Authors’ estimate; Statista^59^
Model 2							
Per-visit drug profit, year 1	69	125	828	3	4	3	Authors’ estimate; personal correspondence^d^
Per-visit drug profit, year 2	97	179	1159	3	4	3	Authors’ estimate; personal correspondence^d^
Per-visit drug profit, year 3	116	207	1391	3	4	5	Authors’ estimate; personal correspondence^d^
Per-visit dispensing fee, year 1	69	125	828	4.09	10.50	15.20	Authors’ estimate
Per-visit dispensing fee, year 2	97	179	1159	4.09	10.50	15.20	Authors’ estimate; Medicaid.gov^60^
Per-visit dispensing fee, year 3	116	207	1391	4.09	10.50	15.20	Authors’ estimate; Medicaid.gov^60^
Per-visit incentive pay, year 1^e^	69	125	828	30	40	50	Authors’ estimate including key informants
Per-visit incentive pay, year 2^e^	97	179	1159	20	30	45	Authors’ estimate including key informants
Per-visit incentive pay, year 3^e^	116	207	1391	11	20	40	Authors’ estimate including key informants

Abreviations: NA, not applicable; OTP, opioid use disorder.

^a^ PertBeta distribution was used for all inputs with minimum and maximum values listed.

^b^ All monetary amounts are shown in 2024 US dollars.

^c^ Monthly rent was applied only for the base case in model 1, as it was excluded in the scenario analysis; see additional details in eTable 6 in Supplement 1.

^d^ M. Olivier, PharmD, via notes after a telephone call, June 16, 2024.

^e^ Incentive pay was applied only to the base case in model 2; it was excluded in the scenario analysis.

#### Anticipated Clientele and Visit Intensity

To estimate the client base for model 1, we assumed a shift of 10% to 13% of long-term OTP methadone-receiving clients to the pharmacy-based medication unit and assumed a modest market growth equivalent to between 8% and 10% of the US population who have both an OUD and perceived need for treatment^55^ (eTable 3 in Supplement 1). We further assumed a 40% growth in clients for year 2 and a 20% growth for year 3 (Table 1)^37,40,41,42,43,44,45,46,47,48,49,50,51,52,53,54,55,56,57^ to simulate a strong ramp-up period and then leveling-off period of recruitment. Visit intensity varied between 1 and 28 visits per patient-month for model 1 and between 1 and 4 visits for model 2.

#### Startup Costs

Startup costs for model 1 included wages for training time for key pharmacy staff (Table 1^37,40,41,42,43,44,45,46,47,48,49,50,51,52,53,54,55,56,57^; eTables 4 and 5 in Supplement 1). We included the cost of legal services for OTP and pharmacy partnership contract review (Table 1).^37,40,41,42,43,44,45,46,47,48,49,50,51,52,53,54,55,56,57^ Other program startup costs included the labor to set up parallel methadone dispensing systems, all costs associated with DEA registration, a DEA-approved safe,^43,44,45,46,61^ and a single-drawer locked cabinet for disposal.^40,41,42^ In the scenario analysis for model 1, liquid methadone–related costs, telehealth-related infrastructure, and security personnel costs were added. Model 2 startup costs included staff training and time to set up new standard operating procedures (Table 1).^37,40,41,42,43,44,45,46,47,48,49,50,51,52,53,54,55,56,57^

#### Annual Costs

Annual costs for model 1 included staff wages,^37^ DEA licensing fees,^62^ costs associated with additional training and/or compliance tasks, and alcohol wipes (scenario analysis only). For model 2, annual costs included staff wages,^37^ DEA licensing fees,^62^ ongoing training and/or compliance tasks, and the cost of methadone.^57^ The same overhead percentage range (5%-15%) was applied to all costs in both models.

#### Income Sources

Model 1 and model 2 income calculations were different due to the OTP relationship required by model 1 (Table 2).^58,59,60^ In model 1, we assumed a flat monthly fee of between $1379 and $2068 per month, paid by the OTP to the pharmacy as part of a contractual agreement (Table 2^58,59,60^; eMethods 1 in Supplement 1).^32,37^ We included the fair market rental value^59^ for the equivalent of 18 square feet (eg, to acknowledge space required for the high-security safe and dispensing preparation), paid monthly by the OTP to the pharmacy.

Model 2 income assumed an enhanced fee-for-service payment structure with a per-prescription profit on the methadone, a standard dispensing fee, and an added amount paid by the payer similar to incentive payments already in place for vaccines and buprenorphine administration (Table 2).^58,59,60,63,64^ The incentive payments were stepped down over the 3 years, starting at $40 and ending at $20. Moreover, we assumed a startup grant of $5000 from state or local entities.^65^

The ROI was conducted from a pharmacy perspective for both models. We used Monte Carlo simulation (10 000 iterations), a typical methodology to estimate the most likely outcomes of an uncertain event.^66^ We used a PertBeta distribution for all our inputs with uncertainty ranges because it is a flexible distribution that can handle both normally distributed and skewed data. We conducted a scenario analysis for model 1 with increased costs and decreased income to simulate an OTP partner negotiating a more favorable financial agreement (eTable 6 in Supplement 1).^32,37^ We further conducted a scenario analysis for model 2 to simulate payers not providing incentive payments, along the lines of vaccines in pharmacies. All analyses were conducted using Microsoft Excel via Microsoft 365 (Microsoft Corp) and @Risk, version 7.51 industrial (Lumivero).^67^ All costs are in 2024 US dollars; future costs were discounted by the standard 3% rate.

## Results

### Model 1: Pharmacy-Based Medication Unit

#### Component Findings

##### Client Visits

The number of client visits over the 3 years was 3429 (95% UI, 1385-6244), suggesting a mean (SD) of 95 (35.6) visits per month. No changes were made to this estimate for the scenario analysis.

##### Costs

We estimated a 1-time expense of $4437 (95% UI, $3331-$5714) in startup costs for the pharmacy. For the scenario analysis, startup costs totaled $7221 (95% UI, $5399-$9717). Including all costs together gave a total of $38 152 (95% UI, $19 788-$64 505) in the base model and $72 278 (95% UI, $34 480-$130 171) in the scenario analysis.

##### Revenue

Total revenue over the 3 years (including the $5000 startup grant) was $135 057 (95% UI, $39 267-$320 105). For the model 1 scenario analysis, total revenue was lower at $117 436 (95% UI, $22 270-$301 934).

#### Return on Investment

Over 3 years, $3.53 (95% UI, $1.14-$6.99) was returned to a participating pharmacy for every $1.00 spent (Table 3). This translated into a 3-year net profit of $96 904 (95% UI, $5365-$267 451), including a 93.8% likelihood of netting $15 000 by year 3 (Figure, A). For the scenario analysis in which costs were increased and income was decreased, $1.61 (95% UI, $0.37-$3.43) was returned for every $1.00 spent (Table 3). The top 3 most influential inputs associated with these findings were the monthly payments, the number of visit intensity for year 3, and the visit intensity for year 2; the rest of the top 10 inputs and additional results are shown in eFigures 1 to 4 in Supplement 1. Scenario analysis showed a 25.3% chance of loss (eFigure 4A in Supplement 1).

**Table 3.** Pharmacy ROI for Pharmacy-Based Medication Unit and Pharmacist-Dispensed Methadone Models After 3 Years

Model	ROI for each $1 invested, $ (95% uncertainty interval)^a^
1	3.53 (1.14-6.99)
1-SA	1.61 (0.37-3.43)
2	2.64 (2.04-3.41)
2-SA	1.09 (0.85-1.39)

Abbreviations: ROI, return on investment; SA, scenario analysis.

^a^ All monetary amounts are shown in 2024 US dollars.

**Figure.**
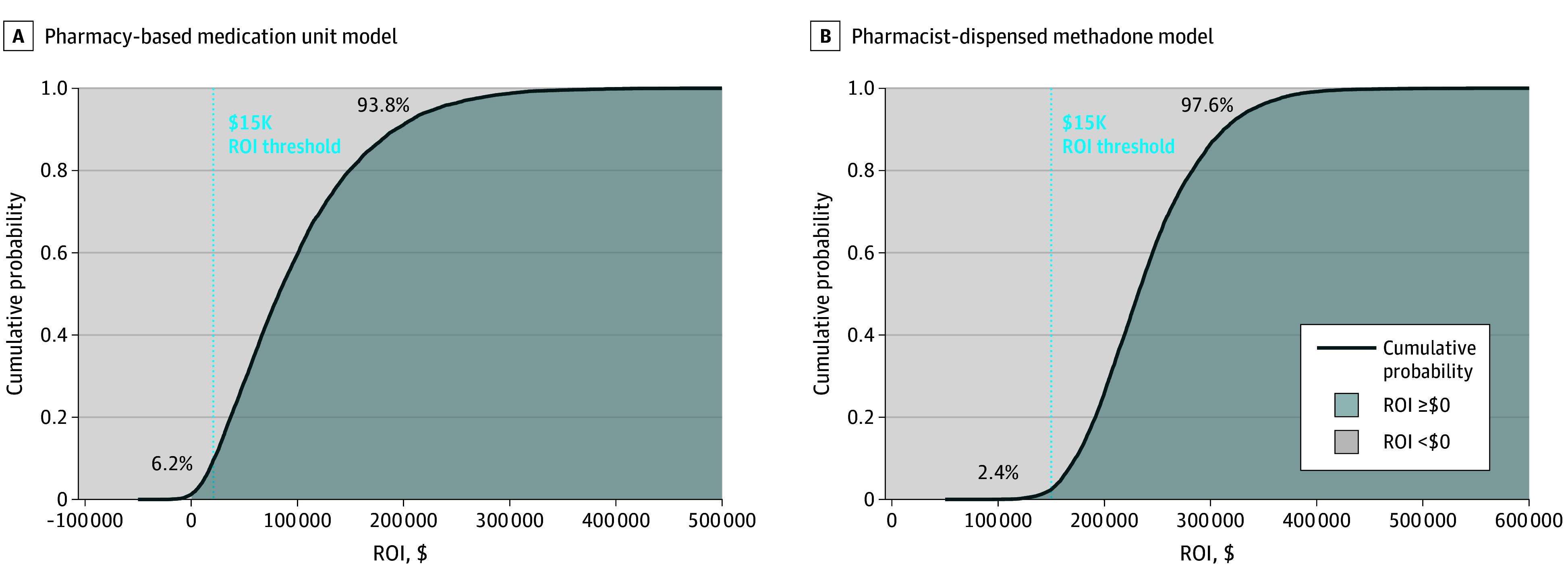
Cumulative Probability Curve of Pharmacy Return on Investment (ROI) After 3 Years

### Model 2: Pharmacist-Dispensed Methadone

#### Component Findings

##### Client Visits

The number of client visits over the 3 years was 793 (95% UI, 531-1167). This suggests a mean (SD) of 22 (4.9) visits per month.

##### Costs

Pharmacy startup costs were $1435 (95% UI, $793-$2389). Three-year costs totaled $14 703 (95% UI, $10 370-$20 799).

##### Revenue

Including the $5000 startup grant, total revenue was $38 547 (95% UI, $27 014-$55 075). For the model 2 scenario analysis, in which revenue was reduced by eliminating per-visit incentive payments, the total revenue was $15 895 (95% UI, $11 971-$21 524).

#### Return on Investment

Over 3 years, $2.64 (95% UI, $2.04-$3.41) was returned to a participating pharmacy for every $1.00 spent (Table 3). This translated into a 3-year net profit of $23 844 (95% UI, $15 045-$36 546), including a 97.6% likelihood of netting $15 000 or more by year 3 (FigureB). The scenario analysis showed a return of $1.09 (95% UI, $0.85-$1.39) for every $1.00 spent. The 3 most influential inputs associated with both these ROIs were the hourly wage of the pharmacist, the year 3 pharmacist time per visit, and the year 2 pharmacist time per visit; the rest of the top 10 and additional findings are shown in eFigures 5 to 7 in Supplement 1. Scenario analysis showed a 25.9% chance of loss (eFigure 7 in Supplement 1).

## Discussion

We estimated a positive ROI for pharmacies participating in a pharmacy-based medication unit (model 1) and in the pharmacist-dispensed methadone model (model 2), demonstrating a high probability of profitability for pharmacies. However, the model 1 scenario analysis showed a 25.3% chance of loss (eFigure 4A in Supplement 1), and the model 2 scenario analysis showed a 25.9% chance of loss (eFigure 7 in Supplement 1). Thus, pharmacy leaders can use these parameters to guide contract negotiations to better ensure profitability. Methadone is a highly effective medication for OUD^2^ that, to date, has generally been available only through OTPs.^1,4^ OTPs alone cannot address the need for methadone treatment, especially in rural areas.^23,26^ People with OUD taking methadone need additional ways to receive their treatment that are more accessible, convenient, and normalized.^17^ Pharmacies are well suited to address this community need, but until now, financial pathways to expand in this way have been unclear.

The pharmacy-based medication unit is permissible under federal law, and previous research has shown this model’s feasibility.^68^ Several barriers to its implementation exist, including unclear and cumbersome regulations requiring the methadone for OUD to be delivered to the pharmacy by the OTP and stored and managed in a system separate from all other controlled substances at the pharmacy.^4^ Absent these requirements, several of the costs in our model would decrease to zero, suggesting even greater pharmacy ROI, and potentially some reduced costs from the OTP perspective.

The pharmacy-based medication unit necessitates contractual agreements between OTPs and pharmacies that do not violate the Anti-Kickback Statute, and these agreements are the most influential input in model 1 (eFigure 1 in Supplement 1). Currently, OTPs generally have a monopoly on delivering methadone; partnering with pharmacies would represent a significant change to their business models. More research is needed to understand the financial impact of the pharmacy-medication unit model from the OTP perspective. States could consider using opioid settlement funds to incentivize OTPs and pharmacies to partner together to expand methadone access.^69,70^

The pharmacist-dispensed model represents a significant shift in the delivery of methadone in the US, to a model consistent with how methadone is dispensed globally and in US peer countries.^71,72,73,74^ People receiving methadone for OUD in the US have called for the implementation of this type of model, as have prescribers who feel alignment with other medications for addiction treatment is appropriate and overdue.^17,22^ Despite the evidence base supporting adoption, interventions for access to medications for opioid use disorder are limited in their reach due to policy barriers that delay or restrict care and leave access inconsistent across states.^75^ The results of the analyses are supportive of the DEA and Substance Abuse and Mental Health Services Administration expanding certification for methadone dispensing in pharmacies.

Nationwide, pharmacies are struggling to stay open; closures have disproportionately affected independent pharmacies, those in rural areas, and those serving low-income neighborhoods.^76,77,78^ In the current landscape, pharmacy owners may not be willing to diversify or expand their services without positive financial forecasting. The results of this economic analysis can support pharmacies in making informed decisions about the financial risks involved in expanding their business model.^79^

State actors can make participating in pharmacy-based methadone more attractive to pharmacy owners by implementing tiered dispensing fees in which differential reimbursement rates are determined based on pharmacy location and prescription volume.^80,81^ States could also support pharmacy-based methadone by recognizing pharmacists as clinicians, which would enable them to expand the clinical services they offer and receive payment from health insurance plans for those services.^79,82,83^

### Limitations

Our study has important limitations. First, our study required that we make several assumptions in the absence of publicly available data needed to perform an ROI analysis. The analysis could be more or less accurate depending on the accuracy or implementation of any given assumption. Our analysis was informed by preferences expressed by people with lived experience accessing methadone who participated in our community advisory board meetings or the Liberating Methadone Conference; these preferences do not necessarily represent all people with OUD, and there may be additional requirements from this community that are essential to implementing pharmacy-based methadone. Furthermore, some people experience OTPs as punitive and unaccommodating.^16,84^ People may hesitate to use the pharmacy-based medication unit approach because of the ongoing involvement of the OTP; however, some people perceive this as a welcomed improvement even if the OTP is still involved.^20^

Although our analysis draws on a combination of key informant interviews, the literature, and observed pharmacy operations, specific startup and operational costs may not fully capture the variability in costs across different pharmacies, although our findings remained robust in the scenario analysis that increased costs. State policies governing medication units differ and may change the financial projections modeled in this study. In the pharmacist-dispensed methadone model, we assumed states and payers would provide pharmacies with a modest startup grant and other financial incentives. Not all states may do this; however, opioid settlement funds could also support expanding access to medications for opioid use disorder.^69,70^ In addition, we did not include staff turnover given the short time frame; if key staff turnover occurred, it would require additional training expenses.

Although we heard directly from OTP leaders, our cost-sharing assumptions may seem too generous; OTPs may not agree to pay the monthly rental fee that we included in model 1. To mitigate the potential for overestimating the ROI, we used a Medicaid-rate assumption in the base case, and then we excluded the rental payment and additional expenses in a scenario analysis. As our calculations resulted in a per-pharmacy ROI, forecasting the exact number of participating pharmacies falls outside the scope of this study. Nevertheless, by modeling potential financial implications, we aim to highlight several pathways through which states, OTPs, and pharmacies could expand access to this service. Furthermore, while the ROI on the OTP side for model 1 is beyond the scope of the present study, future research may conduct this analysis to inform OTP expansion of satellite dispensing.

## Conclusions

The results of this economic analysis estimate positive ROIs for pharmacies engaged in either a pharmacy-based medication unit or pharmacist-dispensed methadone model. Pharmacies and OTPs should consider partnerships that expand access to methadone treatment (ie, medication units), and regulators should consider changes that facilitate pharmacist-dispensed methadone.

## Notes

Supplement 1. **eTable 1.** Summary of Model Details and Differentiation **eMethods 1.** Further Methodological Details **eTable 2.** Results of the Pharmacy Time-in-Motion Study **eMethods 2.** Cost of Labor Equations **eTable 3.** Estimating Starting Range of Clients per Participating Pharmacy **eTable 4.** Hours Related to Startup Training Assumptions **eTable 5.** Costs Related to Startup Training Assumptions **eTable 6.** Scenario Analysis for the Pharmacy-Based Medication Unit Model **eFigure 1.** Pharmacy-Based Medication Unit Model Top Ten Inputs Impacting Mean Return on Investment **eFigure 2.** Scenario Analysis, Pharmacy-Based Medication Unit Model Top Ten Inputs Impacting Mean Return on Investment **eFigure 3.** Pharmacy-Based Medication Unit Model 3-Year Net Profit at $50K ROI Threshold **eFigure 4.** Scenario Analysis, Pharmacy-Based Medication Unit Model 3-Year Net Profit at $0K, $15K, $50K ROI Thresholds **eFigure 5.** Pharmacist-Dispensed Methadone Model Top Ten Inputs Impacting Mean Return on Investment **eFigure 6.** Scenario Analysis, Pharmacist-Dispensed Methadone Model Top Ten Inputs Impacting Mean Return on Investment **eFigure 7.** Scenario Analysis, Pharmacist-Dispensed Methadone Model 3-Year Break-Even at $0K ROI Threshold
eReferences.

Supplement 2.
Data Sharing Statement
