# Supplementary material for: Treating Opioid Use Disorder With Methadone in Pharmacies
Source: JAMA Netw Open. 2026 Mar 16;9(3):e260703. doi: 10.1001/jamanetworkopen.2026.0703 (PMC12993698; doi:10.1001/jamanetworkopen.2026.0703)
Supplement: Supplement 1. — eTable 1. Summary of Model Details and Differentiation eMethods 1. Further Methodological Details eTable 2. Results of the Pharmacy Time-in-Motion Study eMethods 2. Cost of Labor Equations eTable 3. Estimating Starting Range of Clients per Participating Pharmacy eTable 4. Hours Related to Startup Training Assumptions eTable 5. Costs Related to Startup Training Assumptions eTable 6. Scenario Analysis for the Pharmacy-Based Medication Unit Model eFigure 1. Pharmacy-Based Medication Unit Model Top Ten Inputs Impacting Mean Return on Investment eFigure 2. Scenario Analysis, Pharmacy-Based Medication Unit Model Top Ten Inputs Impacting Mean Return on Investment eFigure 3. Pharmacy-Based Medication Unit Model 3-Year Net Profit at $50K ROI Threshold eFigure 4. Scenario Analysis, Pharmacy-Based Medication Unit Model 3-Year Net Profit at $0K, $15K, $50K ROI Thresholds eFigure 5. Pharmacist-Dispensed Methadone Model Top Ten Inputs Impacting Mean Return on Investment eFigure 6. Scenario Analysis, Pharmacist-Dispensed Methadone Model Top Ten Inputs Impacting Mean Return on Investment eFigure 7. Scenario Analysis, Pharmacist-Dispensed Methadone Model 3-Year Break-Even at $0K ROI Threshold eReferences. [file jamanetwopen-e260703-s001.pdf]

Supplementary Online Content

Tschampl CA, Feltus SR, Soranno E, et al. Treating opioid use disorder with methadone in pharmacies. *JAMA Netw Open.* 2026;9(3):e260703. doi:10.1001/jamanetworkopen.2026.0703

eTable 1. Summary of Model Details and Differentiation
eMethods 1. Further Methodological Details
eTable 2. Results of the Pharmacy Time-in-Motion Study
eMethods 2. Cost of Labor Equations
eTable 3. Estimating Starting Range of Clients per Participating Pharmacy
eTable 4. Hours Related to Startup Training Assumptions
eTable 5. Costs Related to Startup Training Assumptions
eTable 6. Scenario Analysis for the Pharmacy-Based Medication Unit Model
eFigure 1. Pharmacy-Based Medication Unit Model Top Ten Inputs Impacting Mean Return on Investment
eFigure 2. Scenario Analysis, Pharmacy-Based Medication Unit Model Top Ten Inputs Impacting Mean Return on Investment
eFigure 3. Pharmacy-Based Medication Unit Model 3-Year Net Profit at $50K ROI Threshold
eFigure 4. Scenario Analysis, Pharmacy-Based Medication Unit Model 3-Year Net Profit at $0K, $15K, $50K ROI Thresholds
eFigure 5. Pharmacist-Dispensed Methadone Model Top Ten Inputs Impacting Mean Return on Investment
eFigure 6. Scenario Analysis, Pharmacist-Dispensed Methadone Model Top Ten Inputs Impacting Mean Return on Investment
eFigure 7. Scenario Analysis, Pharmacist-Dispensed Methadone Model 3-Year Break-Even at $0K ROI Threshold
eReferences.

This supplementary material has been provided by the authors to give readers additional information about their work.

© 2026 Tschampl CA et al. *JAMA Network Open.*

eTable 1. Summary of Model Details and Differentiation

Model	Description	Revenue for pharmacies	Visits
**Model 1**	Medication Unit in Pharmacies Strategy; OTPs & pharmacies form relationships; permissible under current law	▪ Flat monthly rate paid from OTP to pharmacy hosting the Med Unit<br>▪ Additional flat monthly "rent" fee	5-15 starting clients per month; 1-28 visits per month; mix of observed and take-home doses
**Model 2**	Pharmacist-Dispensed Methadone Strategy; Federal legislation or regulation changes required	▪ Profit on medicine<br>▪ Dispensing fee<br>▪ Incentive fee from Medicaid/state	6-17 starting clients per month; 1-4 visits per month; take-home doses only

Abbreviation: OTPs = opioid treatment programs.

© 2026 Tschampl CA et al. *JAMA Network Open.*

eMethods 1. Further Methodological Details

Additional qualitative methodological details

To identify key components required for pharmacy-based methadone models we obtained perspectives from people who use methadone in several ways. First, we reviewed extant sources where experiences accessing methadone and pharmacy-based methadone delivery have been discussed. Specifically, this included review of the Liberating Methadone Conference Report (1) and conference recordings from which we identified key considerations that applied to both models (e.g., need for access at locations other than OTPs, pharmacists training in OUD treatment, and a private space for observed dosing).

Second, following the COBRE Principles for Community Empowering Research (2), we conducted two in-person community advisory board (CAB) meetings in June 2024 with individuals with current and former experience accessing methadone for OUD treatment (n=8). The first CAB meeting was held at a community-based research organization and included people in long-term recovery from opioid use disorder with experience accessing methadone through the OTP system (n=3). The second meeting took place at a local harm reduction drop-in center and convenience sampling was used to recruit people in early recovery from OUD and/or current drug users who had experience with methadone or buprenorphine treatment. After obtaining verbal consent, we briefly presented an outline of the medication unit model and the possibilities of a pharmacist-dispensed model to participants. We asked participants open ended questions about preferred dosing schedules, pharmacy hours, privacy considerations, services they would want to see offered at the pharmacy, stigma, and infrastructure considerations. We allowed time for participants to express other components of pharmacy-based methadone they would want to be available if they were able to receive methadone for OUD at the pharmacy. Meetings were audio recorded and lasted 60 minutes and participants were compensated ($40 cash). A team member deidentified and transcribed the audio recording verbatim to facilitate identification of additional components relevant to both models, including a desire for pharmacy staff to undergo anti-stigma training, and not requiring urine toxicology testing at the pharmacy.

To further identify startup and operational costs to include in the models, we conducted interviews with opioid treatment program leadership (n=5), pharmacy leadership (n=7), private and public payers (n=6) and state and federal policymakers (n=5). We used a combination of purposive and snowball sampling to recruit individuals. Interviews took place from April to July 2024 via videoconference for 40-60 minutes. Participants provided verbal consent, and interviews were recorded and transcribed. Participants were presented with an outline of the medication unit model and the pharmacist-dispensed model, informed with assumptions and components identified in the Liberating Methadone Conference Report and CAB meetings once those data were collected.

We searched the literature and commercial websites to extract values for cost and revenue inputs not specifically obtained through interviews. We used the Bureau of Labor Statistics to ascertain staff wages. (3) To help calculate labor costs, we conducted a time-motion study,(4) simulating tasks relevant to one or both models while recording and timing the tasks. Cost of labor equations are displayed in eMethods 2. Finally, we obtained final specific values through a consultation with an independent pharmacy owner.(5) Please note, none of the interviews were managed by a potential methadone distribution market participant (i.e., not by a pharmacy chain, pharmacy benefit manager, or distributor).

© 2026 Tschampl CA et al. *JAMA Network Open.*

Additional details regarding quantitative methodology

We combined the data we collected with micro-costing methodology (6) to estimate cost and revenue values not directly received from key informants or the literature.

Anticipated clientele and visit intensity: To produce a reasonable starting clientele range, we began with a thought experiment described in eTable 3. Then, to reflect a maturing client base we calibrated the proportion of new-to-the-pharmacy clients to start at 70% and decreased by 20% each year for years 2 and 3.

The visit intensity for Model 1 ranged from one to 28 visits per month, to allow for a mix of daily and weekly visits with some monthly visits. The visit intensity for Model 2 was less than that for Model 2 because the typical pattern for most prescriptions is monthly. Therefore, we narrowed the monthly visit range from one to four in Model 2. Also, for Model 2, we assumed the convenience to the client entices slightly more people to demand the service, so we increased the clientele estimate from Model 1 by 15%. For both models, we allowed the pharmacy size to vary between a single pharmacist to four; and no pharmacy techs to 11. We assumed client numbers would stabilize after year three.

Startup costs. Startup costs for Model 1 included wages for pharmacist time to train in OTP procedures (e.g., documentation, reporting, ordering, inventory management) and anti-stigma training for pharmacy staff (Table 1 and eTables 4 and 5). We included the cost of legal services for OTP-pharmacy contract review (Table 1). Other program startup costs included the labor to set up a parallel ordering and tracking system for methadone; all costs associated with DEA registration; a DEA-approved safe (7–12); and a single-drawer locked cabinet for disposal (13–15). In the scenario analysis, telehealth-related infrastructure was added, as well as training for a certified security staff. Model 2 startup costs included time to set up new standard operating procedures, training on methadone maintenance treatment and anti-stigma for pharmacists, and pharmacy technicians (Table 1).

Annual costs: Annual costs for Model 1 included staff wages (3), DEA licensing fees (16), costs associated with maintaining a parallel documentation and management system, medication costs, and sanitation supplies. For Model 2, annual costs included staff wages (3) and reoccurring costs such as DEA licensing fees (16), and the cost of methadone.(17) Staff time to meet ongoing training/regulatory requirements are in both models, as is the same, flat overhead percentage (e.g., lights, internet, etc.) applied to all startup and annual costs. A list of cost-related inputs is shown in Table 1.

Different methadone modalities were considered in the different models. Model 1 included only diskettes; the scenario analysis included diskettes and liquid methadone. Model 2 included pill and diskette forms. Therefore, the staff time it takes to prepare doses varies. The results of the authors' time-in-motion study are shown in eTable2 and represent the basis for the ranges used in the modeling (Table 1).

We considered translation services for clients who prefer to receive their health information in a language other than English, but the most likely value was zero for the entire estimated cost range. This is because we assumed large chain pharmacies to have a call-in service subscription already and small independent pharmacies to have an existing call-in service, have a technician that speaks a language common to their client population, and/or use an existing translation app on their smart phone.

© 2026 Tschampl CA et al. *JAMA Network Open.*

Additionally, we considered the wholesale cost of ordering the methadone to distribute, but one of our key informants provided a range of profit levels. Therefore, we only needed to include those profit levels (Table 2) in the revenue list rather than in both the costs and revenue lists.

Income sources: Model 1 and Model 2 income calculations were different due to the OTP relationship required by Model 1 (Table 2). In Model 1, we assumed a flat monthly fee paid by the OTP to the pharmacy as part of a profit-sharing agreement (Table 2 and eTable 6). The flat monthly fee was built around Medicare reimbursement rate to OTPs for the drug-portion of a methadone treatment payment bundle (i.e., $40.71) (18) reduced by 30% to simulate Medicaid rates (the more common among clients of methadone maintenance therapy) multiplied by the expected number of stable monthly clients (i.e.,15) and adjusted +/- 20% for a probable range of $1,368-$2,068 per month.

We included the fair market rental value (19) for the equivalent of 18 square feet (e.g., to acknowledge space required for the high-security safe and dispensing preparation), paid monthly by the OTP to the pharmacy for the base case.

Of note, The Anti-Kickback Statute (AKS) is a criminal law that prohibits offering, paying, soliciting, or receiving any form of remuneration to induce or reward referrals or business involving items or services reimbursable by federal healthcare programs.(24) Although rent paid to the pharmacy for medication unit space by the OTP is considered remuneration, the law details several ways to make these rental agreements meet “safe harbor” (i.e., not subject to AKS) requirements.(25)

Similarly, safe harbor can be achieved through a profit-sharing agreement between the OTP and the pharmacy with a medication unit that does not connect profits to patient volume or referrals. We achieved this by creating a flat monthly payment that does not vary by patient volume or referrals. Finally, state or local anti-kickback laws may also apply and be more strict than federal law. These should be reviewed prior to forming partnerships.

Model 2 income assumed an enhanced fee-for-service payment structure with a per-prescription profit on the methadone, a standard dispensing fee, and an added amount paid by the payer similar to incentive payments already in place for vaccines and buprenorphine administration.(20,21) The incentive payments are varied along a range and decreased over the three years, starting at an average of $40 per visit and ending at $20. Moreover, we assumed a startup grant of $5,000 from state or local entities (Table 2).(22)

The return on investment was conducted from a pharmacy perspective for both models. We used Monte Carlo simulation (10,000 iterations), a typical methodology to predict the most likely outcomes of an uncertain event. (23) Monte Carlo simulation uses random sampling to simulate a system or process, in this case, integrating methadone dispensing at pharmacies for treatment of opioid use disorder. By running the simulation through 10,000 iterations we build a robust distribution of the likely return on investment if a pharmacy undertook this business activity (i.e., methadone dispensing). Monte Carlo simulation is particularly useful for scenarios with high levels of uncertainty or complexity, and these return-on-investment (ROI) analyses include both.

Monte Carlo simulation allowed us to vary all these 121 inputs simultaneously and probabilistically along a Pert-Beta distribution, which we chose for all the probabilistic inputs given its ability to handle skewed data (as well as normally distributed data).

© 2026 Tschampl CA et al. *JAMA Network Open.*

We conducted a scenario analysis for Model 1 with increased costs and decreased income to simulate an OTP partner negotiating a more favorable financial agreement (Supplement eTable 6). We further conducted a scenario analysis for Model 2 to simulate payers not providing incentive payments along the lines of vaccines in pharmacies. Despite the evidence base supporting incentive payments for encouraging the spread of a new public health intervention, severe budget cuts for Medicaid passed in 2025 increase the probability of this scenario.

All analyses were conducted using Microsoft Excel and Palisade @Risk (https://www.palisade.com/risk/). All costs were adjusted to 2024 US dollars; future dollars were discounted by the standard 3% rate.

© 2026 Tschampl CA et al. *JAMA Network Open.*

eTable 2. Results of the Pharmacy Time-in-Motion Study

Staff type	Task	10mg pills/90 mg	10mg pills/40 mg	10mg pills/ 220mg	40mg disk/ 90 mg	40mg disk/ 40mg	40mg disk/ 220mg	90mg liquid	40mg liquid	220mg liquid
PTECH	Greet & ID check	0.3	0.3	0.3	0.3	0.3	0.3	0.3	0.3	0.3
PHARM	Set up	0.5	0.2	1.3	0.5	0.2	1.2	1.1	0.5	2.8
PHARM	Count 1 days' worth	0.5	0.2	1.2	0.1	0.1	0.3	1.0	0.5	2.5
PHARM	C2 Documentation (paper)	0.4	0.4	0.4	0.4	0.4	0.4	0.4	0.4	0.4
PHARM	Create label & PDMP-like data entering x 2	2.5	2.5	2.5	2.5	2.5	2.5	2.5	2.5	2.5
PTECH	Place label x1	0.1	0.1	0.1	0.1	0.1	0.1	0.1	0.1	0.1
PHARM	Clean up	0.1	0.1	0.1	0.1	0.1	0.1	0.2	0.2	0.2
PHARM	Filling lock box	0.2	0.2	0.2	0.2	0.2	0.2	0.2	0.2	0.2
PTECH	Check out process	0.5	0.5	0.5	0.5	0.5	0.5	0.5	0.5	0.5
PTECH	Add to system, etc.	1.0	1.0	1.0	1.0	1.0	1.0	1.0	1.0	1.0
PHARM	Counseling	7.4	7.4	7.4	7.4	7.4	7.4	7.4	7.4	7.4
PTECH	Greet, bring to privacy area, check ID	1.0	1.0	1.0	1.0	1.0	1.0	1.0	1.0	1.0
PHARM	Set up	0.5	0.2	1.3	0.5	0.2	1.2	1.1	0.5	2.8
PHARM	Count 1 days' worth	0.5	0.2	1.2	0.1	0.1	0.3	1.0	0.5	2.5
PTECH	C2 Documentation (paper)	0.4	0.4	0.4	0.4	0.4	0.4	0.4	0.4	0.4
PHARM	PDMP-like data enteringx2	0.5	0.5	0.5	0.5	0.5	0.5	0.5	0.5	0.5
PTECH	Print label	0.7	0.7	0.7	0.7	0.7	0.7	0.7	0.7	0.7
PTECH	Place label	0.1	0.1	0.1	0.1	0.1	0.1	0.1	0.1	0.1
PHARM	Observe consumption	1.1	0.5	2.8	1.1	0.5	2.8	0.6	0.3	1.4
PHARM	Clean up	0.1	0.1	0.1	0.1	0.1	0.1	0.2	0.2	0.2
PTECH	Check out process	0.5	0.5	0.5	0.5	0.5	0.5	0.5	0.5	0.5
PTECH	Add to electronic system, etc.	1	1	1	1	1	1	1	1	1
PHARM	Counseling	7.4	7.4	7.4	7.4	7.4	7.4	7.4	7.4	7.4

Notes: Time is expressed in minutes: All data were recorded at an independent pharmacy, during a time-in-motion study conducted by the authors.

© 2026 Tschampl CA et al. *JAMA Network Open.*

eMethods 2. Cost of Labor Equations

General equations used for calculating the annual cost of labor:

(1) *Annual cost for labor type j = (wage$_j$ / hour)* (hours$_j$ / patient visits) * (visits/ year)*

(2) *Annual cost for labor type j = (wage$_j$ / hour)* (hours$_j$ /year)*

© 2026 Tschampl CA et al. *JAMA Network Open.*

eTable 3. Estimating Starting Range of Clients per Participating Pharmacy

To estimate the starting range of clients for our pharmacy return on investment models, we began with a thought experiment regarding how some clients would shift from OTPs and some new clients would join, enticed by the ability to receive methadone at a community pharmacy. For the "new" clients, we drew from the documented number of people with a substance use disorder who perceive a need for treatment, reduced to those with opioid use disorder, and then a small percentage of those (8-10%). We then estimated the number of participating pharmacies and divided the total range of clients by the estimated range of participating pharmacies. Thus, we arrived at the starting point range of 5-15 clients for the base case of the medication unit in a pharmacy strategy (Model 1). For subsequent years, we estimated 40% growth in year 2 of operation to simulate pharmacies' work to build their clientele, and 20% growth in year 3 of operations, at which point the clientele would level off. We increased this clientele range by 15% for the pharmacist-dispensed methadone strategy (Model 2) based on the assumption of a greater number of registered providers allowed to prescribe MMT to be filled at the pharmacy.

Item	Min	Best	Max	References
Total methadone maintenance treatment clients at OTPs	n/a	378,787	n/a	(26)
% shift to pharmacy	10%	11%	13%	Authors' estimate
N shift to pharmacy for MMT	37,879	41,667	49,242	Authors' calculation
N people with SUD plus perceived need for treatment	n/a	2,089,000	n/a	(26)
% of these with OUD	n/a	11%	n/a	Authors' estimate
N people with OUD plus perceived need for treatment	n/a	229,790	n/a	Authors' calculation
% of these newly starting MMT	8%	9%	10%	Authors' estimate
N of people newly starting MMT	18,383	20,681	22,979	Authors' calculation
N MMT clients (shifted + new)	56,262	62,348	72,221	Authors' calculation
Estimated distribution of MMT clients among pharmacies	12,343	6,789	4937	Authors' calculation, (27,28)
N starting MMT clients per participating pharmacy	9	5	15	Authors' calculation

Abbreviations: Best = the best estimate parameter for PertBeta distribution; max = maximum value for PertBeta distribution; min = minimum value for the PertBeta distribution; MMT = methadone maintenance treatment; OUD = opioid use disorder; SUD = substance use disorder.

© 2026 Tschampl CA et al. *JAMA Network Open.*

eTable 4. Hours Related to Startup Training Assumptions

Training need	Model 1			Model 1 SA			Model 2 and 2 SA			Citations
	Hrs per staff	Hrs (min)	Hrs (max)	Hrs per staff	Hrs (min)	hrs (max)	Hrs per staff	Hrs (min)	Hrs (max)	
Anti-stigma training (Pharmacist)	2	1.5	4	2	1.5	4	4	1.5	4	(29–31)
Anti-stigma training (PTech)	2	1	8	2	1	8	4	1	8	(29–31)
Parent OTP procedures (Pharmacist)	4	3	7	4	3	7	n/a	n/a	n/a	Author's estimate; (32)
How to treat OUD training (Pharmacist)	n/a	n/a	n/a	n/a	n/a	n/a	4	4	10	(33,34)
Motivational interviewing (Pharmacist)	n/a	n/a	n/a	n/a	n/a	n/a	n/a	n/a	n/a	(35)
SA, Telemedicine equipment (PTech)	n/a	n/a	n/a	0.5	0.25	1	n/a	n/a	n/a	(36)

Note: Types of training to include in the analyses were chosen based on key informant interviews. Abbreviations: Hrs = hours; max = maximum value; min = minimum value; n/a = not applicable; PTech = pharmacy technician; SA = scenario analysis.

© 2026 Tschampl CA et al. *JAMA Network Open.*

eTable 5. Costs Related to Startup Training Assumptions

Training need	Cost for training (best)	Minimum cost	Maximum cost	Citations
Anti-stigma training (Pharmacist)	0	0	20	(29–31)
Anti-stigma training (PTech)	0	0	20	(29–31)
Parent OTP procedures (Pharmacist)	0	0	0	OTP provides the training
How to treat OUD training (Pharmacist)	0	0	125	(33)
Motivational interviewing[a] (Pharmacist)	0	0	0	Authors' estimate
SA, Telemedicine equipment[a] (PTech)	0	0	0	Authors' estimate

[a]There are many free options for both motivational interviewing training and for learning how to use new telemedicine-related equipment and software. Types of training were chosen based on key informant interviews.
Abbreviations: n/a = not applicable; OTP = opioid treatment programs; PTech = pharmacy technician; SA = scenario analysis.

© 2026 Tschampl CA et al. *JAMA Network Open.*

eTable 6. Scenario Analysis for the Pharmacy-Based Medication Unit Model

For the scenario analysis (SA) related to the pharmacy-based medication unit model, we focused on additional provisions the partner OTP may negotiate to produce a more favorable contractual agreement for itself, e.g., the pharmacy purchases liquid methadone dispensing equipment and lower total monthly payments. This allowed us to test the robustness of the positive ROI for pharmacies found in the Model 1 base case.

Input item	Justification	Added for SA	Subtracted from base case for SA
Monthly “rental” payment from OTP to pharmacy	Testing the robustness of the ROI finding in the base case.		X
Telemedicine equipment and software	OTPs may improve patient-centered care if adding an option for clients to receive counseling immediately before/after receiving methadone at the pharmacy.	X	
Training on new equipment for pharmacy technician	Companion to the immediately previous item.	X	
Liquid methadone dispensing machine and time to dispense	OTPs can provide different forms of methadone. We assumed partnering OTPs and pharmacies would want to keep costs the lowest, and so we focused the base case on diskettes and added liquid to the SA only.	X	
OTP bottle-labeling machine	In the event an OTP imposed strict branding requirements, a separate labeling machine would be required.	X	
Training and wages for a certified security guard	A DEA-required safe is included in the base case, but pharmacies all have security systems in place (some with physical guards). Interviewees all said they would not need additional security measures; therefore, reserved for this scenario analysis.	X	

Abbreviations: DEA = Drug Enforcement Agency; OTP = opioid treatment program; ROI = return on investment; SA = scenario analysis.

© 2026 Tschampl CA et al. *JAMA Network Open.*

eFigure 1. Pharmacy-Based Medication Unit Model Top Ten Inputs Impacting Mean Return on Investment

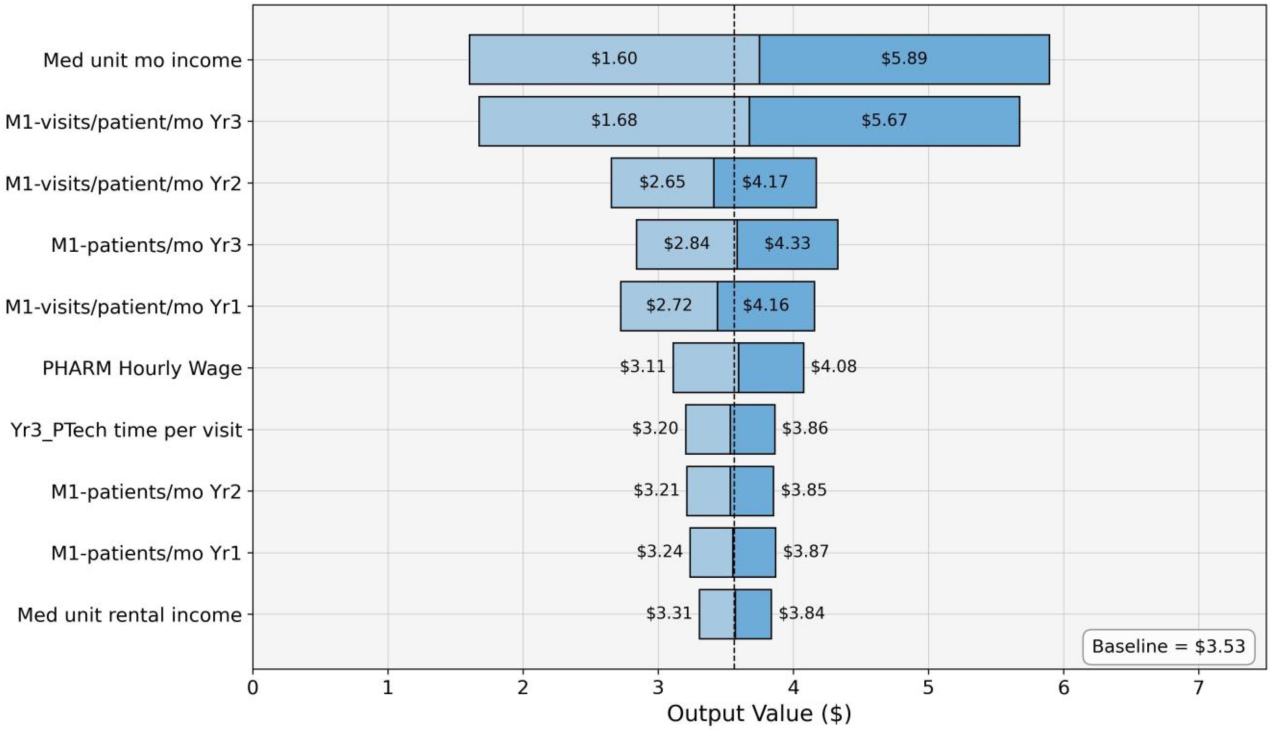

Abbreviations: M1 = Model 1; med unit = medication unit; mo = month; PHARM = pharmacist; PTech = pharmacy technician; Yr = year.

© 2026 Tschampl CA et al. *JAMA Network Open.*

eFigure 2. Scenario Analysis, Pharmacy-Based Medication Unit Model Top Ten Inputs Impacting Mean Return on Investment

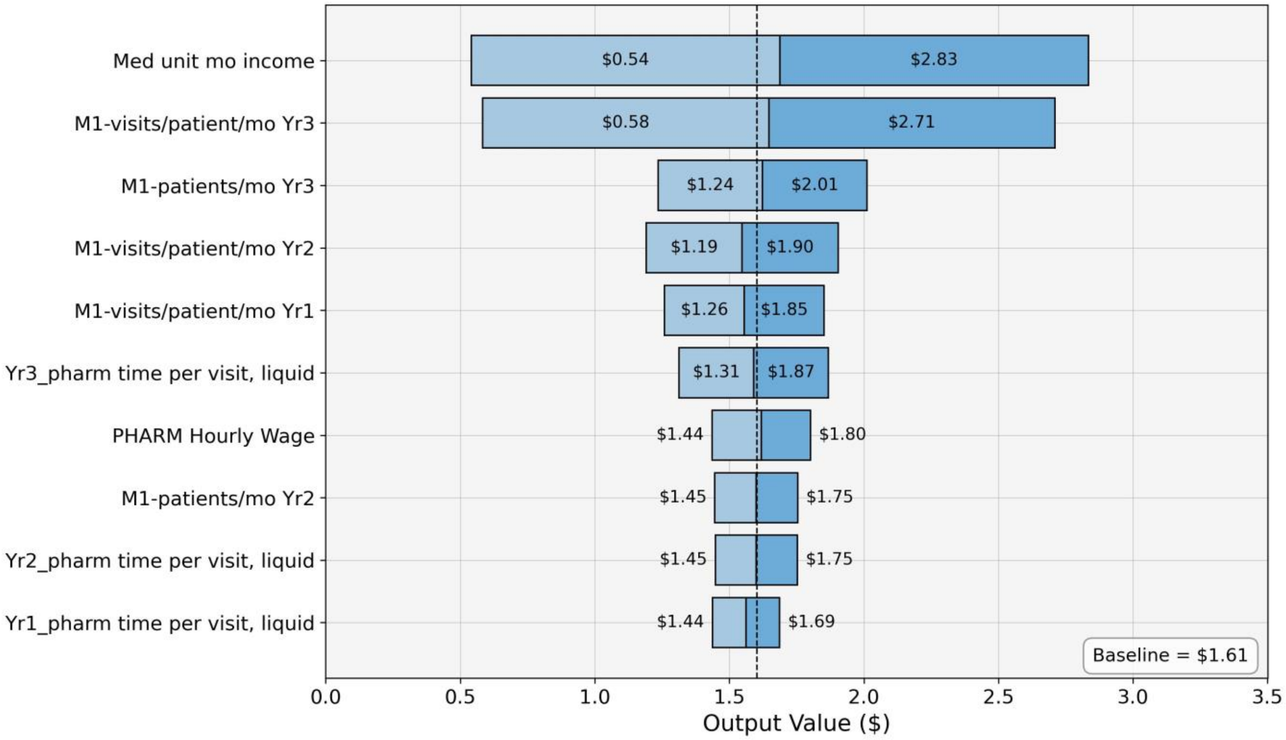

Abbreviations: SA = scenario analysis; liquid = methadone in liquid form included in the assumption; M1 = Model 1; mo = month; Yr = year.

© 2026 Tschampl CA et al. *JAMA Network Open.*

eFigure 3. Pharmacy-Based Medication Unit Model 3-Year Net Profit at $50K ROI Threshold

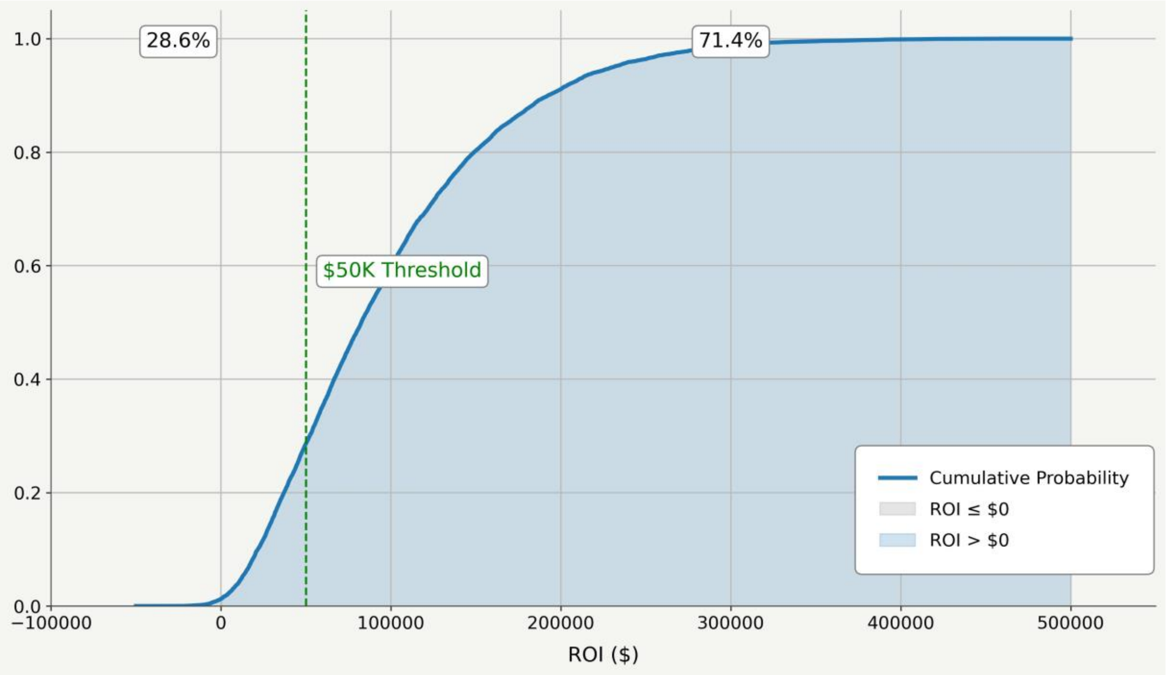

Abbreviations: K = thousand; ROI = return on investment.

© 2026 Tschampl CA et al. *JAMA Network Open.*

eFigure 4. Scenario Analysis, Pharmacy-Based Medication Unit Model 3-Year Net Profit at $0K, $15K, $50K ROI Thresholds

Panel A: $0K profit threshold

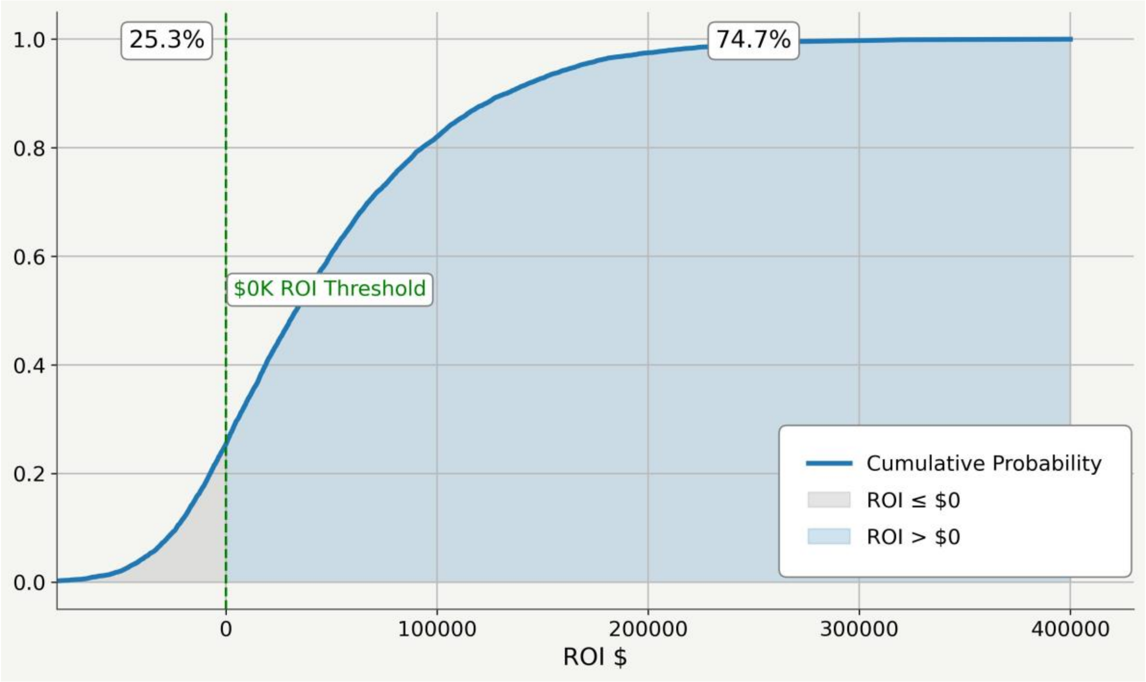

Panel B: $15K profit threshold

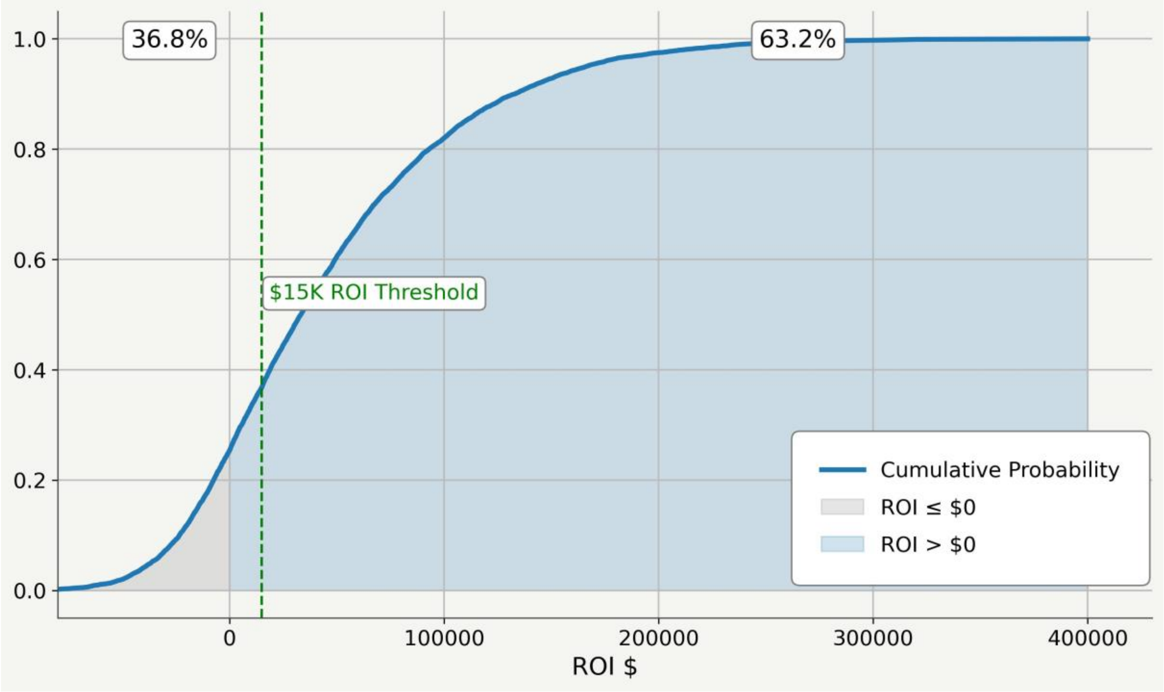

© 2026 Tschampl CA et al. *JAMA Network Open.*

Panel C: $50K profit threshold

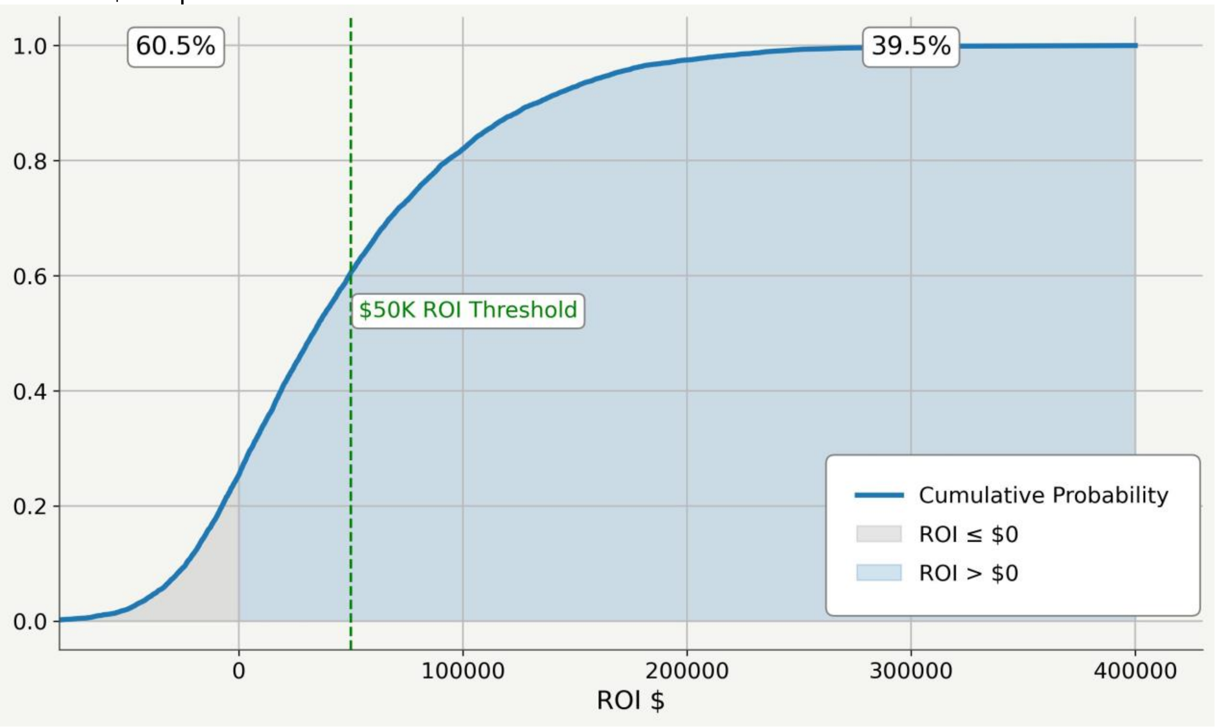

Abbreviations: K = thousand; ROI = return on investment.

© 2026 Tschampl CA et al. *JAMA Network Open.*

eFigure 5. Pharmacist-Dispensed Methadone Model Top Ten Inputs Impacting Mean Return on Investment

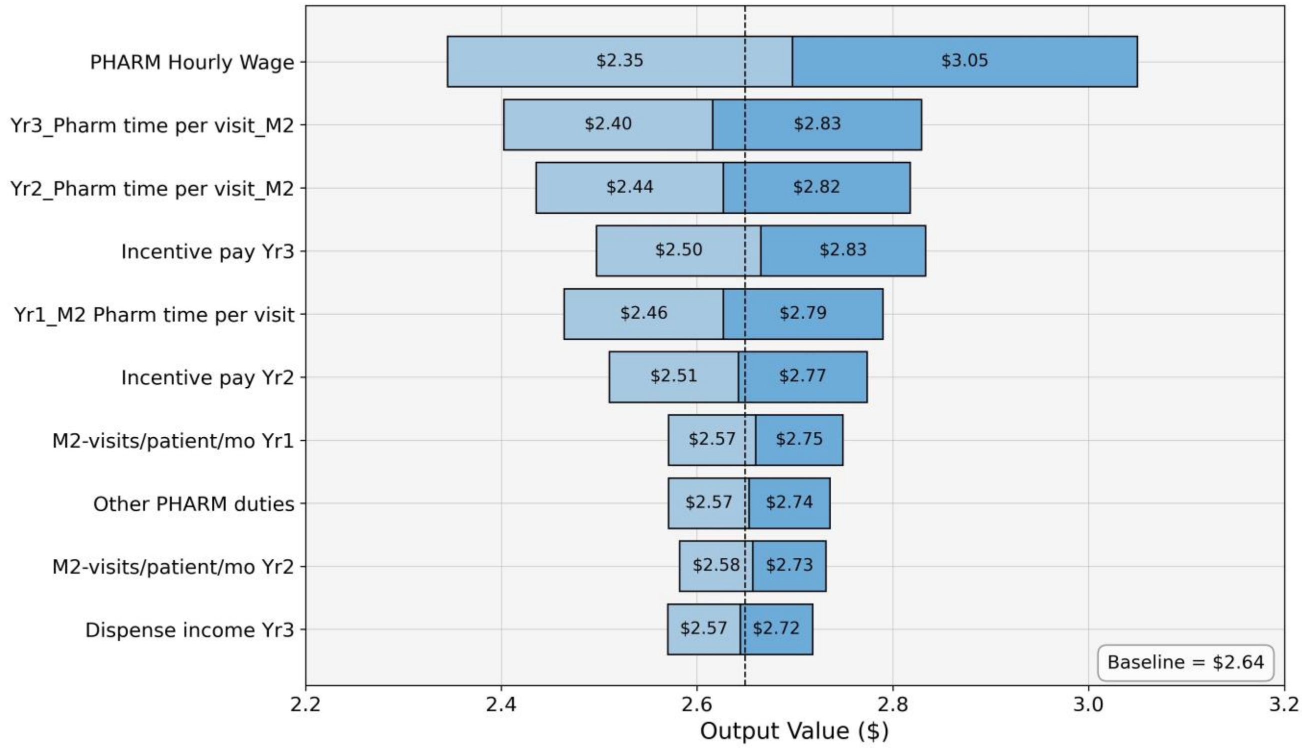

Abbreviations: d = day; disk = methadone in diskette form; M2 = Model 2; mo = month; Pharm = pharmacist; Yr = year.

© 2026 Tschampl CA et al. *JAMA Network Open.*

eFigure 6. Scenario Analysis, Pharmacist-Dispensed Methadone Model Top Ten Inputs Impacting Mean Return on Investment

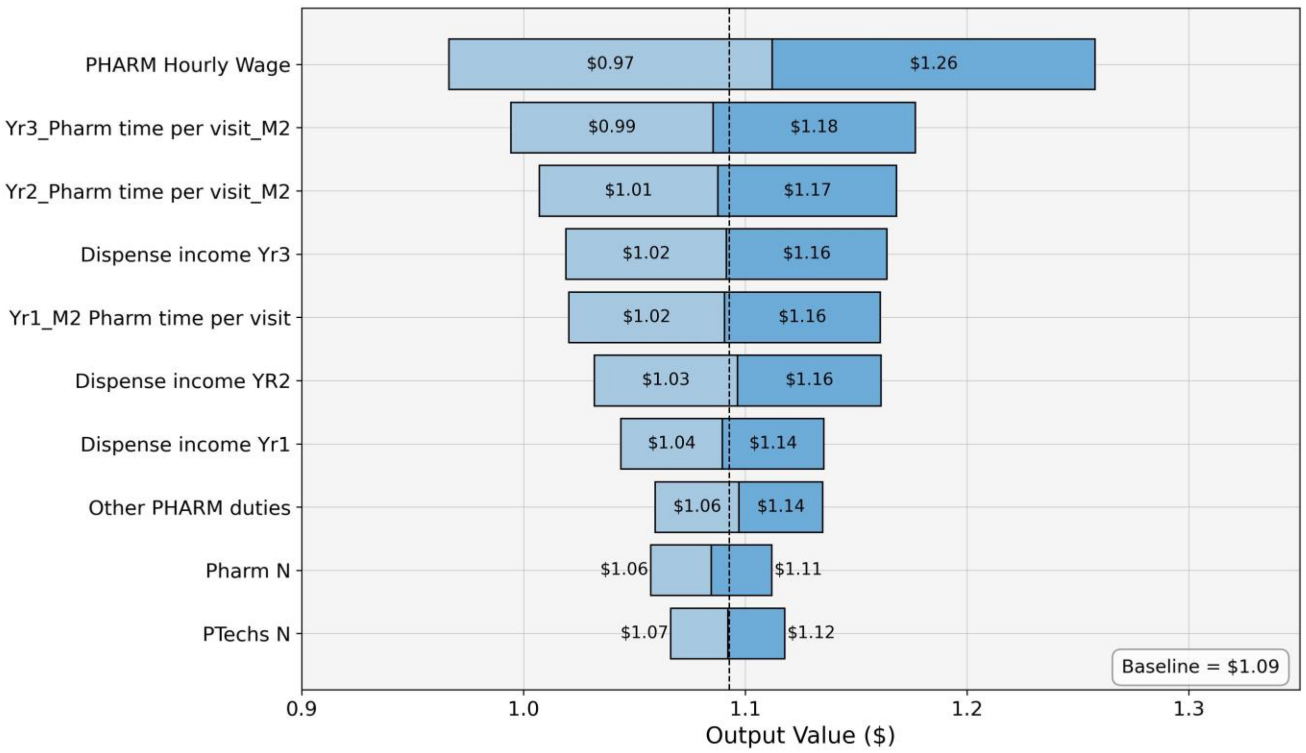

Abbreviations: M2 = Model 2; mo = month; Pharm = pharmacist; Yr = year.

© 2026 Tschampl CA et al. *JAMA Network Open.*

eFigure 7. Scenario Analysis, Pharmacist-Dispensed Methadone Model 3-Year Break-Even at $0K ROI Threshold

Panel A: $0K profit threshold

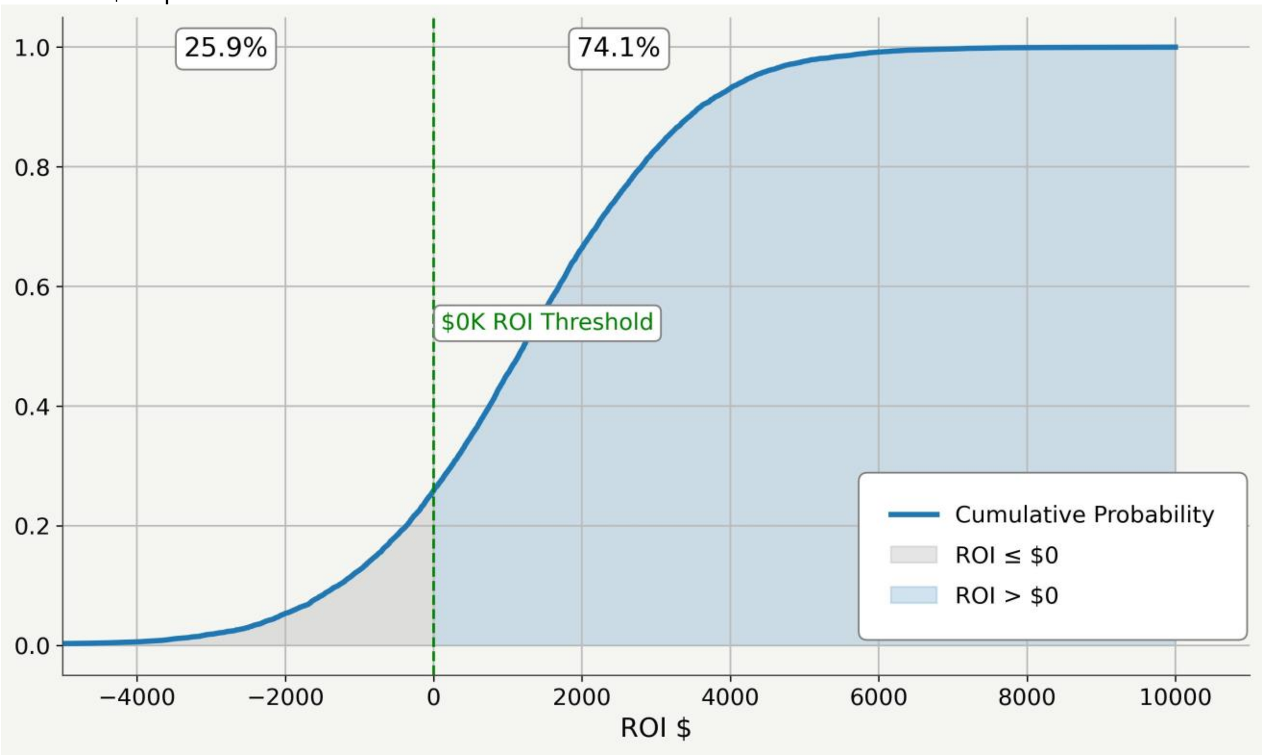

© 2026 Tschampl CA et al. *JAMA Network Open.*

eReferences.

1. National Coalition to Liberate Methadone, National Survivors Union, NYU Langone Center for Opioid Epidemiology and Policy. Liberating Methadone: A Roadmap for Change Conference Proceedings and Recommendations [Internet]. New York, NY; 2024 June [cited 2025 Feb 3]. Available from: https://drive.google.com/file/d/1eRwpgfpfuR9Miphk5b7BsHRs4TEeYOBC/view?usp=sharing

2. Community Engaged Research Core of the COBRE on Opioids and Overdose at Rhode Island Hospital. Shaking the Tree of Science: COBRE's Principles for Community Empowering Research. 2022 Apr.

3. U.S. Bureau of Labor Statistics. 2023 [cited 2025 June 5]. Available from: https://www.bls.gov/oes/2023/may/oes_stru.htm

4. Kalne PS, Mehendale AM. The Purpose of Time-Motion Studies (TMSs) in Healthcare: A Literature Review. Cureus. 2022;14(10):e29869.

5. Personal Correspondence with Matt Olivier, PharmD. 2024.

6. Frick KD. Micro-Costing Quantity Data Collection Methods. Med Care. 2009 July;47(7 Suppl 1):S76.

7. Amazon.com. Grafco Locking Medicine Cabinet - Safe Narcotic, Drug & Medical Storage - Steel Double Doors & Locks [Internet]. [cited 2025 June 5]. Available from: https://www.amazon.com/Grafco-Locking-Narcotic-Various-Sizes/dp/B000FYZC68?th=1

8. Graham-Field. Grafco Locking Medicine Cabinet - Safe Narcotic, Drug & Medical Storage - Steel Double Doors & Locks : Home & Kitchen [Internet]. [cited 2025 June 5]. Available from: https://www.amazon.com/Grafco-Locking-Narcotic-Various-Sizes/dp/B000FYZC68?th=1

9. Uline. Digital Safe - Floor, Fire Rated, 21 x 20 x 29 [Internet]. [cited 2025 June 5]. Available from: https://www.uline.com/Product/Detail/H-8939/Safes/Digital-Safe-Floor-Fire-Rated-21-x-20-x-29-4-0-Cu-Ft

10. Global Industrial. Global Industrial™ Medical Security Cabinet w/Double Key Locks, 8"W x 2-5/8"D x 12-1/8"H, Beige [Internet]. [cited 2025 June 5]. Available from: https://www.globalindustrial.com/p/global-174-medical-security-cabinet-8-w-x-2-5-8-d-x-12-1-8-h-2-key-locks-sand

11. Global Industrial. Global Industrial™ Small Narcotics Cabinet, Double Door/Double Lock, 12"W x 8"D x 15"H, Beige [Internet]. [cited 2025 June 5]. Available from: https://www.globalindustrial.com/p/global-industrial-153-small-narcotics-cabinet-double-door-double-lock-12-w-x-8-d-x-15-h-beige

12. 21 CFR Part 1301 - Security Requirements [Internet]. Available from: https://www.ecfr.gov/current/title-21/part-1301/subject-group-ECFRa7ff8142033a7a2

13. Litfad. File Cabinet Metal Locking Drawers Plain Vertical File Cabinet - 1.1mm 5 White [Internet]. [cited 2025 June 5]. Available from: https://www.litfad.com/file-cabinet-metal-locking-drawers-plain-vertical-file-cabinet-s-2864162.html?srsltid=AfmBOortKeycfTQJzHwhqPy9m6MEl1bTHaAtsIwAm03UBXlFcXvzjfvA

14. Global Industrial. Global Industrial™ Stacking Steel Drawer, 17-1/4"W x 20"D x 12"H, Black [Internet]. [cited 2025 June 5]. Available from: https://www.globalindustrial.com/p/stacking-drawer-12-h

15. Uline. Collaboration Mobile Pedestal File - 2-Drawer, White H-8260W [Internet]. [cited 2025 June 5]. Available from: https://www.uline.com/Product/Detail/H-8260W/Office-Storage/Collaboration-Mobile-Pedestal-File-2-Drawer-White

16. Drug Enforcement Administration. Diversion Control Division, Registration [Internet]. [cited 2025 June 5]. Available from: https://www.deadiversion.usdoj.gov/drugreg/registration.html

© 2026 Tschampl CA et al. *JAMA Network Open.*

17. Amazon.com. 400 count, Care Touch Alcohol Wipes Individually Wrapped - Prep Pads with 70% Isopropyl Alcohol [Internet]. [cited 2025 June 5]. Available from: https://www.amazon.com/dp/B06XS38XH6?_encoding=UTF8&ref=cm_sw_r_cp_ud_dp_V7S5KCYZ18F7FW6BXGRW&ref_=cm_sw_r_cp_ud_dp_V7S5KCYZ18F7FW6BXGRW&social_share=cm_sw_r_cp_ud_dp_V7S5KCYZ18F7FW6BXGRW&skipTwisterOG=1.&th=1

18. Centers for Medicare & Medicaid Services. OTP Payment Rates [Internet]. 2024 [cited 2025 June 5]. Available from: https://www.cms.gov/medicare/payment/opioid-treatment-programs-otp/billing-payment/otp-payment-rates

19. Statista. U.S. retail rent by property type 2023 [Internet]. 2023 [cited 2025 June 6]. Available from: https://www.statista.com/statistics/1379047/retail-real-estate-rent-by-property-type-usa/

20. Director Department of Medical Assistance Services, the Commonwealth of Virginia. Increased Reimbursement of Medications for the Treatment of Opioid Use Disorder [Internet]. 2022 [cited 2025 June 5]. Available from: https://vamedicaid.dmas.virginia.gov/memo/increased-reimbursement-medications-treatment-opioid-use-disorder

21. Centers for Medicare & Medicaid Services. Medicare COVID-19 Vaccine Shot Payment [Internet]. 2025 [cited 2025 June 5]. Available from: https://www.cms.gov/medicare/payment/covid-19-vaccine-toolkit/medicare-covid-19-vaccine-shot-payment

22. American Pharmacists Association. APhA Foundation. [cited 2025 June 16]. Incentive Grants. Available from: https://www.aphafoundation.org/incentive-grants

23. Mooney CZ. Monte Carlo Simulation [Internet]. SAGE Publications, Inc.; 1997 [cited 2025 June 11]. Available from: https://methods.sagepub.com/book/mono/monte-carlo-simulation/toc

24. HHS Office of Inspector General. Fraud & Abuse Laws [Internet]. 2021 [cited 2025 Aug 22]. Fraud & Abuse Laws. Available from: https://oig.hhs.gov/compliance/physician-education/fraud-abuse-laws/

25. 42 CFR 1001.952 - Exceptions [Internet]. Available from: https://www.ecfr.gov/current/title-42/part-1001/section-1001.952

26. Substance Abuse and Mental Health Services Administration. Key Substance Use and Mental Health Indicators in the United States: Results from the 2023 National Survey on Drug Use and Health [Internet]. Center for Behavioral Health Statistics and Quality; 2024. Report No.: PEP24-07–021. Available from: https://www.samhsa.gov/data/report/2023-nsduh-annual-national-report

27. Carpenter D, Shubel C, Marley G, Thorpe C, Ostrch B. Community pharmacist opinions about methadone access: Implications for medication units and the modernizing opioid treatment access act. AMERSA; 2025 Nov 15; Portland, Oregon.

28. Berenbrok LA, Tang S, Gabriel N, Guo J, Sharareh N, Patel N, et al. Access to community pharmacies: A nationwide geographic information systems cross-sectional analysis. J Am Pharm Assoc. 2022;62(6):1816-1822.e2.

29. National Association of Boards of Pharmacy. Medication Treatment Opioid Use Disorder [Internet]. Initiatives. [cited 2025 June 17]. Available from: https://nabp.pharmacy/initiatives/medication-treatment/

30. Mountain Plans ATTC. Addressing Stigma and Substance Use Disorders: A HealtheKnowledge Course [Internet]. 2020 [cited 2025 June 17]. Available from: https://attcnetwork.org/addressing-stigma-and-substance-use-disorders-a-healtheknowledge-course/

31. The University of Texas at Austin Dell Medical School. Reducing Stigma Education Tools (ReSET) [Internet]. [cited 2025 June 17]. Available from: https://vbhc.dellmed.utexas.edu/

© 2026 Tschampl CA et al. *JAMA Network Open.*

32. Brooner RK, Stoller KB, Patel P, Wu LT, Yan H, Kidorf M. Opioid treatment program prescribing of methadone with community pharmacy dispensing: Pilot study of feasibility and acceptability. Drug Alcohol Depend Rep. 2022 May 16;3:100067.

33. CDC. Overdose Prevention. 2025 [cited 2025 June 17]. Training: Assessing and Addressing Opioid Use Disorder. Available from: https://www.cdc.gov/overdose-prevention/hcp/trainings/assessing-and-addressing-opioid-use-disorder-oud.html

34. Providers Clinical Support System. 8-Hour MOUD Education Options [Internet]. [cited 2025 June 17]. Available from: https://pcssnow.org/medications-for-opioid-use-disorder/8-hour-moud-education-options/

35. Olmstead TA, Yonkers KA, Forray A, Zimbrean P, Gilstad-Hayden K, Martino S. Cost and cost-effectiveness of three strategies for implementing motivational interviewing for substance misuse on medical inpatient units. Drug Alcohol Depend. 2020 Sept 1;214:108156.

36. VSee Video Demos & Tutorials for BVC [Internet]. VSee. 2020 [cited 2025 June 17]. Available from: https://vsee.com/tutorials/bvc/

© 2026 Tschampl CA et al. *JAMA Network Open.*
