# Supplementary material for: Treating Opioid Use Disorder With Methadone in Pharmacies
Source: JAMA Netw Open. 2026 Mar 16;9(3):e260703. doi: 10.1001/jamanetworkopen.2026.0703 (PMC12993698; doi:10.1001/jamanetworkopen.2026.0703)
Supplement: Supplement 2. — Data Sharing Statement [file jamanetwopen-e260703-s002.pdf]

# Data Sharing Statement

Tschampl. Treating Opioid Use Disorder With Methadone in Pharmacies. *JAMA Netw Open*. Published March 16, 2026. doi:10.1001/jamanetworkopen.2026.0703

**Data**
**Data available:** Yes
**Data types:** Data (not involving human participants)
**How to access data:** tschampl@brandeis.edu
**When available:** With publication

**Supporting Documents**
**Document types:** None

**Additional Information**
**Who can access the data:** researchers whose proposed use of the data has been approved
**Types of analyses:** any purpose
**Mechanisms of data availability:** with a signed data access agreement
